# Animal husbandry and environmental conditions are associated with cefotaxime-resistant *Escherichia coli* in yard soil in peri-urban Malawi

**DOI:** 10.1371/journal.pgph.0006264

**Published:** 2026-07-13

**Authors:** Emma Budden, Caitlin G. Niven, Benjamin Clark, Emily Floess, Blessings Chirwa, Monica Matekenya, Stella Cadono, John Chavula, Victor Chisamanga, Aubrey Dzinkambani, Chisomo Kaponda, Neema Ngondo, Norah Patterson, Sheena Symon, Brighton Austin Chunga, Rochelle H. Holm, Petros Chigwechokha, Francis L. de los Reyes, Cassandra L. Workman, Angela Rose Harris, Ayse Ercumen

**Affiliations:** 1 Department of Plant and Microbial Biology, North Carolina State University, Raleigh, North Carolina, United States of America; 2 Division of Translational Toxicology, National Institute of Environmental Health Sciences (NIEHS), Research Triangle Park, Durham, North Carolina, United States of America; 3 Department of Civil, Construction, and Environmental Engineering, North Carolina State University, Raleigh, North Carolina, United States of America; 4 Department of Water and Sanitation, Mzuzu University, Mzuzu, Malawi; 5 Northern Region Water Board, Mzuzu, Malawi; 6 Department of Biological Sciences, Malawi University of Science and Technology, Limbe, Malawi; 7 Center for Healthy Air Water and Soil, Christina Lee Brown Environme Institute, School of Medicine, University of Louisville, Louisville, Kentucky, United States of America; 8 Department of Anthropology, University of North Carolina Greensboro, Greensboro, North Carolina, United States of America; 9 Department of Forestry and Environmental Resources, North Carolina State University, Raleigh, North Carolina, United States of America; University of Oxford, UNITED KINGDOM OF GREAT BRITAIN AND NORTHERN IRELAND

## Abstract

Soil is an important reservoir for antimicrobial resistance (AMR) and increasingly recognized as a pathogen transmission pathway, especially for young children. However, drivers of domestic AMR soil contamination in low-income countries remain unidentified. We conducted a cross-sectional study with 237 peri-urban households in southern Malawi to identify household and environmental factors associated with cefotaxime*-*resistant *E. coli* in yard soil. Enumerators employed structured surveys and sampled 900 cm^2^ of yard soil per household. We enumerated cefotaxime-resistant *E. coli* in soil using IDEXX Quanti-Tray/2000 with Colilert-18 and cefotaxime supplement, and assessed associations with household sanitation, animal ownership and management, child health and antibiotic use, and weather. Among children <5 years, 25–90% played, ate, slept, or crawled on the ground outside. Of 233 soil samples, 69% harbored cefotaxime-resistant *E. coli* at a mean of 0.90 log_10_ most probable number (MPN) per dry gram*.* Compared to households without animals, household soil had approximately 0.50-log lower mean cefotaxime-resistant *E. coli* concentration if animals were enclosed at night and 0.40-log higher concentration if they were not (p-values<0.05). Mean cefotaxime-resistant *E. coli* concentrations were approximately 0.90-log lower if soil was dry at the time of collection, 0.70-log lower if the household was in the top wealth quintile (p-values<0.005), and 0.30-log lower if any child in the household used antibiotics in the last four weeks (p-value = 0.05). There were no associations with daytime animal confinement, household sanitation, temperature, and ambient humidity. Findings suggest that animal husbandry and soil moisture had stronger associations with cefotaxime-resistant *E. coli* in soil compared to sanitation or antibiotic use, underscoring the importance of a One Health approach to AMR that incorporates domestic animals and environmental factors. Given children’s frequent soil contact, our findings also highlight potential AMR acquisition from soilborne pathways. Studies should quantify soilborne AMR exposure and evaluate associations with animal management/enclosure practices.

## Introduction

Antimicrobial resistance (AMR) caused 1.27 million deaths in 2019 [1]. By 2050, AMR is projected to result in 10 million deaths per year [2], while others predict 2 million annual deaths directly from AMR and 8 million deaths related to AMR [3]. Infections caused by antibiotic-resistant organisms (AROs) are increasingly difficult to treat with available antibiotics and are associated with increased risk of death and complications, duration of illness, and healthcare costs [4–7]. Community colonization with AMR is particularly high in low-income countries. In sub-Saharan Africa, the prevalence of intestinal colonization with extended-spectrum beta-lactamase (ESBL) producing *E. coli* ranged from 5 to 59%, where children <5 years in the Central African Republic had the highest prevalence of colonization [8]. Further, a study conducted in 2023 found that 89% of infants in a Kenya hospital were colonized with ESBL-producing bacteria [9]. In many low-income countries, regulations on antibiotic use are limited [8], and clinically-relevant antibiotics are often available without restriction, both for human use and animal husbandry [10]. Frequent antibiotic use is a known driver of AMR, including self-prescribing or over-prescribing by healthcare professionals [11] and therapeutic or sub-therapeutic use for animals [12].

Poor water and sanitation infrastructure, and contaminated environments further facilitate the spread of AROs and antimicrobial resistance genes (ARGs) between reservoirs and hosts via horizontal gene transfer [13–15]. Antibiotic residues, AROs, and ARGs can be shed in human feces and disseminated into the environment in settings with open defecation or inadequate sanitation [16,17]. Similarly, when domestic animals defecate near the dwelling, antibiotic residues, AROs and ARGs from their feces can spread into soil and other environmental compartments [18,19], as domestic animals are frequently administered antibiotics both therapeutically to treat illness and sub-therapeutically to support growth [20,21]. Within environmental compartments, mobile genetic elements can be exchanged between native communities and pathogens deposited via human or animal fecal waste [22,23]. Environmental conditions, such as temperature and precipitation, may affect the proliferation of ARGs [24]. Additionally, while ARGs can spread naturally in the environment, anthropogenic disturbances can add selective pressures [16,25]. For example, ARG exchange between organisms can be triggered by co-selectors, such as heavy metals, pesticides, and microplastics [25].

**Fig 1:**
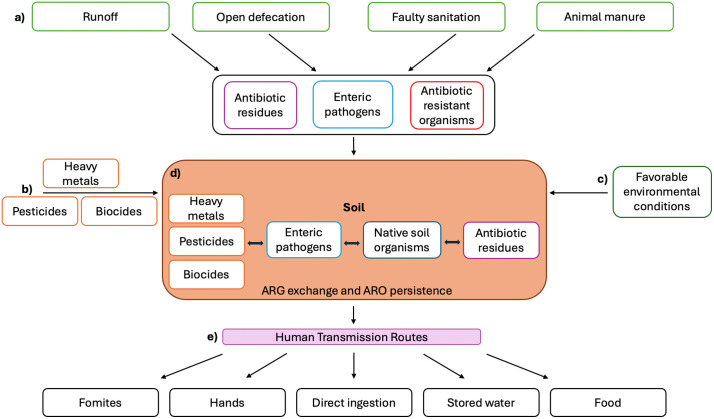
a) Fecal waste streams, including runoff, open defecation, faulty sanitation, and unsafely managed animal manure, can deliver enteric pathogens, antibiotic residues and antimicrobial-resistant organisms (AROs) to soil; b) human-induced disturbances may result in co-selectors, such as heavy metals, pesticides, and biocides in soil; c) environmental variables, such as moisture and elevated temperatures, can support favorable conditions for antibiotic resistance gene (ARG) exchange and ARO persistence; d) native soil organisms interact with delivered pathogens, antibiotic residues, AROs, heavy metals, and chemicals, and a confluence of biological and co-selectors can trigger ARG exchange and support ARO persistence in soil; e) human transmission pathways of AROs from soil include direct ingestion of soil and/or indirect ingestion from soil-contaminated hands, fomites, stored water and/or food.

As one of the most diverse matrices for microbial activity, soil is a particularly important environmental reservoir for AMR [26] because it can offer an efficient environment for ARGs to transfer between soil-inhabited bacteria and clinically-relevant pathogens, such as *E. coli*, *Klebsiella pneumoniae*, and *Salmonella enterica* [27,28] (Fig 1). In rural Bangladesh, a study detected cefotaxime-resistant *E. coli* on 89% of soil floors [29]. Resistance against at least one antibiotic was detected in 42% of *E. coli* isolates from yard soil samples in Bangladesh [30] and 33% of isolates in Peru [31]. Further, a recent study in Nigeria found ESBL-producing *E. coli* in 68% of farm soil samples [32], and a study conducted along a river basin in Tanzania found that 14% of samples positive for *E. coli* harbored ESBL-producing organisms [33].

Soil contaminated with fecal waste can be directly ingested by young children via mouthing behavior or indirectly via contaminated food, water, fomites, and/or hands, which can transfer fecal pathogens to hosts [16,34,35] (Fig 1). Direct soil consumption among children has been correlated with increased diarrheal diseases [36,37]. Early childhood behaviors, such as mouthing soiled hands or objects, further elevates exposure to indirect soilborne routes [38]. In a Monte Carlo simulation utilizing data from rural Bangladesh, 40% of children between six months and two years of age were found to ingest soil [39]. While no studies have assessed whether soil exposure is associated with increased AMR colonization, it is plausible that AROs and ARGs can be transmitted to hosts via soilborne pathways. For example, in a 2022 study in Malawi, having a toilet with a soil floor was associated with higher odds of having ESBL-producing *E. coli* in household members’ stool samples [40].

Sanitation practices have been explored as a determinant of both fecal and AMR contamination in domestic soils, but the evidence is mixed for fecal indicators and scarce for AMR. A study in Tanzania found that having an improved versus unimproved pit latrine was not associated with fecal indicator bacteria in yard soil; soil at the entrance to the home had some of the highest concentrations of fecal indicator bacteria of all sampled locations, including the latrine entrance [41]. It was hypothesized that latrine superstructure may influence this finding because most latrines had open roofs, which may result in bacterial inactivation by sunlight [41]. Similarly, a 2018 study in rural Bangladesh found that having an improved versus unimproved pit latrine was not associated with *E. coli* or ESBL-producing *E. coli* in yard soil [30]. A randomized sanitation intervention providing improved latrines to rural Bangladeshi households did not reduce *E. coli* levels in yard soil; though this study did not evaluate resistance [42]. Sanitation improvements are recommended in AMR control programs [43], and a better understanding of how sanitation infrastructure/practices influence AMR in soil can inform AMR mitigation strategies.

Animal husbandry is a major source of fecal contamination in the environment and plays an important role in disseminating AMR [13]. Hog feces, soil from hog farms, and samples from poultry and cattle farms in various settings have been shown to have high rates of ARG detection, pinpointing animal feces as a source of AMR in animal production facilities [44–48]. Other studies have investigated fecal indicator bacteria and AMR in informal backyard animal husbandry settings, a common practice in low- and middle-income countries where animals are raised within the premises. In Bangladesh, the presence of chickens, cows, goats, and sheep in the household compound increased *E. coli* concentration in yard soil, and chicken ownership in particular was correlated with significantly higher concentration [34]. Also in Bangladesh, cefotaxime-resistant *E. coli* on indoor soil floors had a strong correlation with owning domestic animals and unsafe animal management practices, such as letting animals roam free in the home and keeping them inside the home at night [29]. A recent review found that, in low- and middle-income countries, animals roaming in or near living spaces allows for the exchange of ARGs through environmental compartments, and soil in particular plays a critical role as a mediator in AMR transmission between humans and domestic animals [49]. Given that backyard animal husbandry is a common form of livelihood in low-income countries, the influence of specific animal management practices (e.g., roaming, daytime and nighttime confinement) on the environmental dissemination of AMR needs further study.

Prior research has documented that microbial growth is influenced by environmental and weather factors. Higher soil moisture content promotes proliferation of *E. coli* and ESBL-producing *E. coli* in soil [30]. A 2024 study found a higher prevalence of beta-lactamase genes in soils where manure was applied under higher moisture conditions [50]. Temperature and sunlight also influence *E. coli* concentration in soil [41,51]. A study in rural Tanzania found lower concentrations of fecal indicator bacteria in soil that was in direct sunlight compared to in shade or partial sunlight [41]. Additionally, *E. coli* inoculated into soils from Lake Superior (bordered by the United States and Canada) were found to grow more successfully at temperatures ≥30 °C, with the most growth occurring at 37 °C [51]. Further evidence is needed on the influence of environmental conditions (e.g., sunlight, rainfall, ambient temperature, ambient humidity, soil moisture) specifically on antimicrobial-resistant organisms.

In this study, we aimed to quantify cefotaxime-resistant *E. coli* in yard soil among peri-urban Malawian households. Cefotaxime is a broad-spectrum cephalosporin antibiotic that is commonly used in clinical settings [52] and a known indicator for ESBL-producing bacteria, which are of particular concern because they confer multi-drug resistance against clinically relevant antimicrobials [52]. Specifically, ESBL-producing *E. coli* forms the basis of World Health Organization’s global AMR surveillance in environmental compartments [53]. Here, we used a One Health approach to assess associations between the concentration of cefotaxime-resistant *E. coli* in yard soil and a range of human, animal and environmental factors, including sanitation, animal management practices, antibiotic use by humans and animals, and weather-related variables.

## Methods

### Study design

From June 20 through July 14, 2024, we conducted a cross-sectional study with 237 households in Bangwe, a peri-urban municipality in Blantyre, Malawi. We aimed to enroll households in geographical clusters, corresponding to informal neighborhood delineations [54]. Using ArcGIS, we systematically selected starting points within Bangwe’s geographical boundaries [54], identifying areas central to the community, including churches, schools, mosques, clinics, and shopping hubs. Starting at these points within each targeted area, enumerators travelled in each cardinal direction to screen every third household for eligibility. Households were eligible to enroll if they had a child <5 years old and a caretaker at least 18 years of age consenting to participate.

### Ethical considerations

We fulfilled all Institutional Review Board requirements through Mzuzu University Institutional Review Ethics Committee (#MZUNIREC/DOR/24/38) and the University of North Carolina at Greensboro (UNC-Greensboro IRB #: 20–0245). A caretaker over the age of 18 provided written informed consent to participate in Chichewa. Permission to conduct the study was approved by Blantyre City officials and local leaders in Bangwe township. Additional information regarding the ethical, cultural, and scientific considerations specific to inclusivity in global research is included in the Supporting Information (S1 Checklist).

### Data collection

Enumerators from Mzuzu University and the Malawi University of Science and Technology were trained to administer a structured questionnaire in Chichewa using Open Data Kit (ODK). The questionnaire included questions and spot check observations of household water and sanitation indicators drawn from the Joint Monitoring Programme’s (JMP) Core Questions on Drinking Water, Sanitation and Hygiene for Household Surveys (water source, latrine type and access, child defecation) [55], animal ownership and management (types of domestic animals, daytime and nighttime confinement, antibiotic use), caregiver-reported child health for children <5 years (enteric/respiratory infection symptoms, antibiotic use), environmental factors (sampled soil in sunlight or wet), socio-demographic indicators (assets, expenditures, education, household size, floor material), and caregiver-reported child soil exposure behaviors. If antibiotic use was reported, enumerators asked caregivers for the packaging of any reported antibiotics to confirm. We installed five EXTECH Instruments Humidity/Temperature Dataloggers RHT10 in various locations around Bangwe to record ambient temperature and relative humidity every ten minutes throughout the data collection period. Additionally, we collected publicly available weather data from Weather API, which recorded ambient temperature and humidity every three hours in Blantyre [56], the closest city to Bangwe.

### Soil sample collection

Enumerators trained in aseptic technique identified a courtyard area immediately adjacent to the entrance of the household, avoiding stepping into the sampling area. Two stainless steel corner stencils and a stainless steel spoon were sterilized with 10% bleach and 70% ethanol. The stencils were placed on the soil to measure a 30 x 30 cm area. The spoon was used to scrape the topsoil 10 times vertically and 10 times horizontally and transfer the collected soil into a sterile Whirlpak bag. The Whirlpak bag was placed in a secondary plastic bag to avoid cross-contamination. Samples were transported to the Biological Sciences Laboratory at the Malawi University of Science and Technology in coolers with ice. A field blank was administered every other day. At the first household visited, enumerators opened a Whirlpak bag containing sterile DI water, stirred the sanitized sampling spoon ten times, closed the bag, and returned it to the lab with the soil samples. Due to resource constraints, replicate samples were not collected.

### Enumeration of cefotaxime-resistant *E. coli*

Samples were processed within five hours of collection. The soil sample was homogenized by shaking the Whirlpak bag. Then, 4 g of soil and 40 mL of sterile DI water were added to a 50 mL falcon tube and vortexed for 2 minutes. 10 mL of the homogenized sample was retrieved without letting the soil settle and added to a sterile 100 mL Whirlpak bag containing 90 mL of sterile DI water, creating a 100 mL aliquot that contained 1 g of soil. 80 µL of 5 mg/mL filter-sterilized cefotaxime was added to the 100 mL solution, for a final cefotaxime concentration of 0.004 mg/mL [57], followed by adding Colilert-18 media. Whirlpak bags were gently inverted to mix and, once the media dissolved, the contents were added into a Quanti-Tray/2000, sealed, incubated at 35 °C for 20–22 hours, and visually enumerated for the most probable number (MPN) of cefotaxime-resistant *E. coli* using a UV lamp. To assess moisture content, an approximately 5 g aliquot of soil was weighed to record the wet mass [34], placed into a drying oven at 110 °C, and weighed again after 24 hours to record the dry mass. The moisture content was used to calculate the MPN of cefotaxime-resistant *E. coli* per dry gram of soil.

Field blanks and 10% lab blanks were analyzed using the same procedure as actual samples, using 100 mL of field blank sample and 100 mL of sterile DI water, respectively. Two aliquots were analyzed for each blank, one with and one without cefotaxime. We did not assess samples for generic *E. coli* nor process duplicate samples or positive controls due to resource constraints. However, other studies conducted in low- and middle-income country settings have found a high concentration of generic *E. coli* in soil [29,34,41]. Our enumeration approach was validated in a previous study, which found that the positive control (*E. coli* NC11) used yielded no statistically significant differences in cefotaxime resistant *E. coli* concentration between the IDEXX method and MacConkey plates [57].

### Statistical analysis

We generated a binary variable for the prevalence of cefotaxime-resistant *E. coli* and log_10_-transformed the MPN values per dry gram. Non-detect samples were assigned 0.5 MPN/wet gram of soil (half the lower detection limit) [58], and samples exceeding the upper limit of quantification of 2419 MPN were assigned 2420 MPN/wet gram prior to calculating the MPN/dry gram and taking the logarithm. We tabulated the mean log_10_-transformed MPN of cefotaxime-resistant *E. coli* in yard soil across cross-categories of animal ownership and husbandry practices and cross-categories of latrine type and user load. We used bivariate and multivariable regression to assess associations between household and environmental variables and the log_10_-transformed MPN and prevalence of cefotaxime-resistant *E. coli*. We considered the following independent variables in bivariate models.

#### Sanitation.

We generated binary variables for whether: any feces observed within 2x2 meters of soil sampling area; the household had an improved latrine according to the JMP definition [59]; the household had a flush/pour flush latrine; the latrine was utilized by a single household or shared by multiple households; and children in the household open defecated.

#### Animal ownership and management.

We generated binary variables for whether: the household owned animals; animals were kept in the compound (any animals combined, as well as specific animal types); and any animal feces were observed in the compound. Among households that owned animals, we generated binary variables for whether animals were enclosed during the day and at night, and whether the household gave them antibiotics (to any animals, as well as to specific animal types). Animals were considered enclosed if they were kept in a basket or cage, an animal-only enclosure that they could not leave, or an animal-only enclosure that they could leave freely. Additionally, we generated a categorical variable for animal cohabitation intensity: household owns animals that are not enclosed, household owns animals that are enclosed, household does not own animals.

#### Environmental factors.

We generated binary variables for whether the soil sampling area was in sunlight and whether it was wet at the time of collection as observed by the enumerator. We performed a two-tailed t-test to determine if the enumerator-recorded observation of soil wetness correlated with the sample’s measured moisture content. We tabulated the daily average temperature and humidity using data from the loggers. For study dates where only a subset of loggers was online for 24 hours because of installation/de-installation partway through the day (June 25), we only used data from the loggers that had data for the full 24-hour period. For study dates with no available logger data (June 20, 21, 24), we calculated the daily average temperature and humidity using data from Weather API (weatherapi.com/history/q/blantyre-1598090). We created tertiles of daily average temperature and humidity across the study period and generated binary variables for the top vs. bottom two tertiles.

#### Child illness and antibiotic use.

We generated binary variables for whether any child <5 years in the household had symptoms of caregiver-reported diarrhea, acute respiratory infection (ARI), ARI with fever, or fever in the last seven days, and if any child <5 years in the household used antibiotics in the last four weeks. Diarrhea was defined as 3 or more loose stools in a 24-hour period. ARI was defined as persistent coughing accompanied with shortness of breath, panting, or wheezing [60].

Bivariate models were run for each independent variable. Variables with a p-value of <0.20 in bivariate models were shortlisted for inclusion in multivariable regression models. This broad inclusion criterion was used to ensure that potentially relevant variables were not disregarded. Variables with a p-value <0.05 in multivariable models were considered to have a statistically significant association with the outcome. We estimated variance inflation factors to assess collinearity between weather-related variables (sampling area in sunlight, sampling area wet, average ambient temperature, average ambient humidity) before including them in multivariable models.

Multivariable models also controlled for household’s primary water source, highest education level by any household member, household size, weekly household expenditure, indoor floor material and asset-based household wealth quintile. The water source was categorized according to the JMP definition of improved vs. unimproved [59]. The floor material was categorized as improved (cement/concrete, tile) vs. unimproved (earth/mud). The wealth quintile was calculated using principle component analysis of reported assets [61]. These additional variables were selected for inclusion in multivariable models as potential confounders because household-level water service and socio-demographic factors are expected to influence AMR in the domestic environment [62,63]. Models only included confounders with sufficient variation in the sample (> 5% prevalence in each stratum).

We used generalized linear models to estimate associations. Models with continuous outcomes (log_10_-MPN) used a Gaussian error distribution and identity link to estimate differences in log_10_-transformed MPN, and models with binary outcomes (prevalence) used a Poisson distribution and log link to estimate prevalence ratios. Models accounted for geographic clustering of enrolled households using robust standard errors. All analyses were conducted in R version 4.3.3 through Rstudio version 2024.12.0 + 467. De-identified data and analysis scripts are publicly available at Open Science Framework (https://doi.org/10.17605/OSF.IO/5RG6E).

## Results

### Participant characteristics

We enrolled 237 households and grouped them into 38 independent spatial clusters based on their GPS coordinates [54]. Respondents were an average age of 31.1 years old, and households included an average of 1.2 children <5 years, 1.4 children aged 5–16 years, and 2.7 adults >16 years (S1 Table). The most common education level was secondary (partial or complete), and 87% (205) of respondents reported being able to read and write (S1 Table). Average weekly household expenditure was 17.0 USD (S1 Table). Additionally, 69% (164) of households had electricity in the previous 7 days, 24% (56) owned a refrigerator, 70% (166) owned a mosquito net, and 87% (205) owned a mobile phone (S1 Table).

#### Water and sanitation practices.

84% (200) of households had an improved primary water source, and the most common source was piped water outside the compound (29%, n = 69) (S1 Table). Most households had a latrine, while 19% (46) had an improved latrine, and the most common latrine type was a pit latrine with slab (65%, n = 154) (S1 Table). 71% (168) of respondents reported sharing their latrine with other households, with an average of 11 people per shared latrine (S1 Table). The primary place of defecation for children <5 years was in latrines (40%, n = 95) and nappy/diapers (39%, n = 92) (S1 Table). However, children <5 years were also reported to openly defecate in the compound in 25% (59) of households and outside of the compound in 5% (11) of households (S1 Table). The majority (65%, n = 153) of respondents reported putting/rinsing child feces into the latrine (S1 Table). In 8% (20) of households, fecal waste was observed within 2x2 meters of the soil sampling area in the yard (S1 Table).

#### Animal ownership and management.

Animals were owned by 24% (57) of households; the most common animal was chickens/poultry (16%, n = 37) while other animals included dogs (9%, n = 22), cats (5%, n = 12) and sheep/goats (0.4%, n = 1) (Table 1). Almost three quarters (75%, n = 177) of households reported that animals lived in their compound, where a compound was defined as the land area where the household lives, including extended family members. The most common animal living in the compounds was chickens/poultry (61%, n = 144) while dogs, cats, sheep/goats, and pigs were also reported (Table 1). Households owned an average of 1.8 animals, and an average of 7.1 animals lived in the compound (Table 1). Animals were observed within 2x2 meters of the soil sampling area in the yard in 7% (16) of households, and animal feces were observed in the compound in 37% (21) of households (Table 1).

**Table 1:** Animal ownership and management practices among enrolled households.

Animal ownership	N = 237
Household keeps animals/livestock, % (n)	
Any animal	24.1 (57)
Chickens/poultry	15.6 (37)
Sheep/goats	0.4 (1)
Dogs	9.3 (22)
Cats	5.1 (12)
Other	0.8 (2)
Animals/livestock live in compound, % (n)	
Any animal	74.7 (177)
Chickens/poultry	60.8 (144)
Sheep/goats	2.1 (5)
Pigs	0.4 (1)
Dogs	46.0 (109)
Cats	8.0 (19)
Number of animals kept by household, mean (SD)	
Any animal	1.8 (6.3)
Chickens/poultry	1.5 (6.0)
Sheep/goats	0 (0.1)
Dogs	0.2 (0.7)
Cats	0.1 (0.4)
Other	0 (0.1)
Number of animals living in compound, mean (SD)	
Any animal	7.1 (8.7)
Chickens/poultry	5.0 (6.9)
Sheep/goats	0.1 (0.8)
Pigs	0.2 (3.2)
Dogs	1.7 (2.7)
Cats	0.1 (0.6)
Animals observed within 2x2 meters of soil sampling area, % (n)	6.8 (16)
Animal feces observed in compound, % (n)	36.8 (21)
Animal management practices % (n) ^a^	N = 57 ^b^
Animals kept during the day, % (n)^a^	
Free roaming outdoors	49.1 (28)
Free roaming indoors	47.4 (27)
All enclosed	12.3 (7)
Other	1.8 (1)
Animal ownership among households that enclose animals during the day, % (n)	N = 7 ^c^
Own only chickens/poultry	57.1 (4)
Own chickens/poultry and other animals	28.6 (2)
Own only other animals (sheep/goats, dogs, cats)	14.3 (1)
Animals kept at night, % (n)^a^	
Free roaming outdoors	26.3 (15)
Free roaming indoors	66.7 (38)
All enclosed	21.1 (12)
Other	1.8 (1)
Animal ownership among households that enclose animals at night, % (n)	N = 12 ^d^
Own only chickens/poultry	50.0 (6)
Own chickens/poultry and other animals	41.7 (5)
Own only other animals (sheep/goats, dogs, cats)	8.3 (1)
Household used antibiotics for animals in last 4 weeks, % (n)	N = 57^b^
Any animal	28.1 (16)
Chickens/poultry	14.0 (8)
Dogs	12.3 (7)
Cats	1.8 (1)

^a^Respondent could select multiple answers for these questions.

^b^Asked among 57 households that keep animals.

^c^Asked among 7 households that enclosed animals during the day.

^d^Asked among 12 households that enclosed animals at night.

Among the 57 households that owned animals, during the day animals roamed free outdoors in 49% (28), roamed free indoors in 47% (27), and were enclosed in 12% (7) of households (Table 1). At night, animals roamed free outdoors in 26% (15), roamed free indoors in 67% (38), and were enclosed in 21% (12) of households (Table 1). Among households that used a daytime enclosure, 57% (4) had only chickens/poultry, 29% (2) had chickens/poultry and other animals, and 14% (1) had only other animals, while among households that used a nighttime enclosure, 50% (6) had only chickens/poultry, 42% (5) had chickens/poultry and other animals, and 8% (1) had only other animals (Table 1). In the last four weeks, 28% (16) of households gave antibiotics to any animal in the household, most commonly to chickens/poultry (Table 1).

#### Child illness, antibiotic use and soil exposure.

Among 292 children <5 years in enrolled households, caregivers reported that 16% (48) of children had diarrhea, 46% (134) had an ARI, 24% (70) had an ARI with fever, and 33% (97) had a fever in the last 7 days. In the last four weeks, 33% (97) of children used antibiotics, with an average of 1.2 courses and 1.8 doses of antibiotics taken (Table 2). Of the 97 children who had used antibiotics, the packaging was available for confirming the antibiotic for 17 children. Among these, the packaging matched the reported antibiotic for 76% (13) of children, while for 4 children, the packaging indicated that the reported medication was not an antibiotic. The most common antibiotics were amoxicillin (part of penicillin class) and trimethoprim/sulfamethoxazole (part of sulfonamide class) (Table 2). Antibiotic recommendations most commonly came from doctors (54%, n = 52), followed by self-prescription (29%, n = 28) and pharmacists (17%, n = 16) (Table 2). The most common reason why the child stopped taking antibiotics was that the child felt better (47%, n = 46) (Table 2). In the last three months, caregivers reported an instance of antibiotics being ineffective for 25% (24) of children, and of these, 71% (n = 17) took an additional antibiotic to improve (Table 2). Additionally, among children <5 years, 90% (263) played, 74% (217) ate, 49% (143) slept, and 25% (74) crawled on the ground outside; 9% (27) of children spent no time on the ground outside (Table 2).

**Table 2:** Child health outcomes, antibiotic use and soil exposure.

Health outcomes and antibiotic use	N = 292 ^a^
In the last 7 days, child had, % (n)	
Diarrhea	16.4 (48)
Acute respiratory infection	45.9 (134)
Acute respiratory infection with fever	24.0 (70)
Fever	33.2 (97)
Child used antibiotics in last 4 weeks, % (n)	33.2 (97)
Number of times child used antibiotics in last 4 weeks, mean (SD)	1.2 (3.0)
Last time child used antibiotics	N = 97 ^b^
Number of pills/doses taken, mean (SD)	1.8 (3.2)
Antibiotic used, % (n) ^b^	
Amoxicillin	51.5 (50)
Trimethoprim/sulfamethoxazole	38.1 (37)
Metronidazole/Flagyl	2.1 (2)
Erythromycin	4.1 (4)
Other	9.3 (9)
Who recommended antibiotic use, % (n)	
Doctor	53.6 (52)
Pharmacist	16.5 (16)
Self	28.9 (28)
Other	1.0 (1)
Why did child stop taking antibiotic, % (n) ^c^	
Completed course	35.1 (34)
Antibiotic ran out	27.8 (27)
Child felt better	47.4 (46)
Too expensive	1.0 (1)
Other	6.2 (6)
In the last 3 months, an antibiotic was ineffective, % (n)	24.7 (24)
In the last 3 months, an additional/different antibiotic was taken, % (n) ^d^	70.8 (17)
Child spends time on the ground outside the house (e.g., yard), % (n)	N = 292 ^a^
None	9.2 (27)
Eat	74.3 (217)
Play	90.1 (263)
Sleep	49.0 (143)
Crawl	25.3 (74)

^a^Asked individually for each child <5 years (292 total children).

^b^Asked among the 97 children who used antibiotics at least once in last 4 weeks.

^c^Respondents could select multiple answers.

^d^Asked among the 24 children whose antibiotic was ineffective.

### Cefotaxime-resistant *E. coli* in yard soil

Yard soil samples were collected from 233 of 237 enrolled households; 4 households declined sample collection. Additionally, in 2 samples, we were able to assess the binary prevalence of cefotaxime-resistant *E.* *coli* but could not quantify it because the IDEXX tray did not seal correctly. 69% (160) of samples harbored cefotaxime-resistant *E. coli* at a mean concentration of 0.90 log_10_-MPN/dry gram (standard deviation [SD]=1.1), corresponding to a geometric mean of 8.0 MPN/dry gram (Table 3).

**Table 3:** Soil and environmental characteristics.

Detection of cefotaxime-resistant *Escherichia coli*	N	
Prevalence, % (n)	233 ^a^	68.7 (160)
Log_10_-transformed MPN, mean (SD)	231 ^b^	0.9 (1.1)
Soil at time of collection		
Dry, % (n)	233 ^a^	22.7 (53)
In sunlight, % (n)	233 ^a^	38.6 (90)
Percent soil moisture content		
Sample dry at time of collection, mean (SD)	231 ^b^	4.4 (4.3)
Sample wet at time of collection, mean (SD)	231 ^b^	19.9 (8.0)
Daily average ambient temperature and humidity		
Temperature (°C), mean (SD)	14 ^c^	18.0 (1.1)
Percent relative humidity, mean (SD)	14 ^c^	67.0 (16.1)

MPN: Most probable number, SD = Standard deviation.

^a^Assessed among all soil samples collected.

^b^Assessed among soil samples where cefotaxime-resistant *E. coli* was quantified.

^c^Assessed among dates within the study period.

### Soil and weather conditions

At the time of collection, 39% (90) of soil samples were in sunlight and 23% (53) were dry (Table 3). When the sample was visually wet as reported by enumerators, the measured soil moisture content was significantly higher (20% for wet soil vs. 4% for dry soil, t-test p-value<0.05) (Table 3 and S1 Fig). The daily average temperature was 18.0°C, and the daily average relative humidity was 67% over the study period (Table 3).

### Household and environmental factors associated with cefotaxime-resistant *E. coli* in yard soil

Among cross-categories of animal type and animal enclosure practices, the concentration of cefotaxime-resistant *E. coli* in yard soil was highest among households that owned poultry and did not enclose their animals at night (mean log_10_-MPN: 1.42) and generally appeared higher when animals were not enclosed at night, regardless of the types of animals owned (S2 Fig). Among cross-categories of number of latrine users and latrine type, cefotaxime-resistant *E. coli* concentration in soil was high among households whose latrine was unimproved and used by an above-median (>9) number of people (mean log_10_-MPN: 1.05), as well as among households whose latrine was not flush/pour flush and used by an above-median number of people (mean log_10_-MPN: 1.07). Cefotaxime-resistant *E. coli* concentration in soil generally appeared higher among households where the latrine was used by multiple households. Unless the latrine was used by a single household, households with an unimproved latrine appeared to have higher cefotaxime-resistant *E. coli* levels compared to households with an improved latrine.

In bivariate regression, households had a statistically significant lower concentration of cefotaxime-resistant *E. coli* per dry gram of yard soil if owned animals were reported to be enclosed at night (Δlog_10_: -1.12 (-1.56, -0.67); p-value<0.0005), if the family’s latrine was used by a single household rather than shared with other households (Δlog_10_: -0.42 (-0.72, -0.12); p-value = 0.01), if the soil was dry at time of collection (Δlog_10_: -0.92 (-1.29, -0.56); p-value<0.0005), or if the ambient temperature was in the top tertile (Δlog_10_: -0.31 (-0.59, -0.03); p-value = 0.03) (S2 Table). While not statistically significant, households also appeared to have lower concentration of cefotaxime-resistant *E. coli* if they had an improved latrine, soil was in sunlight at time of collection, or if children used antibiotics in the last four weeks (S2 Table). On the other hand, households appeared to have a higher concentration of cefotaxime-resistant *E. coli* in yard soil if the household owned chickens/poultry (S2 Table). The following variables were not associated with cefotaxime-resistant *E. coli* concentration in yard soil: utilizing a flush/pour flush latrine, children in household openly defecating, feces or animals observed near sampling area, owning animals, owning dogs/cats, compound keeping animals, animals enclosed during the day, household giving antibiotics to any animal, ambient humidity, and children having diarrhea, ARI, fever, or ARI with fever (S2 Table).

**Fig 2:**
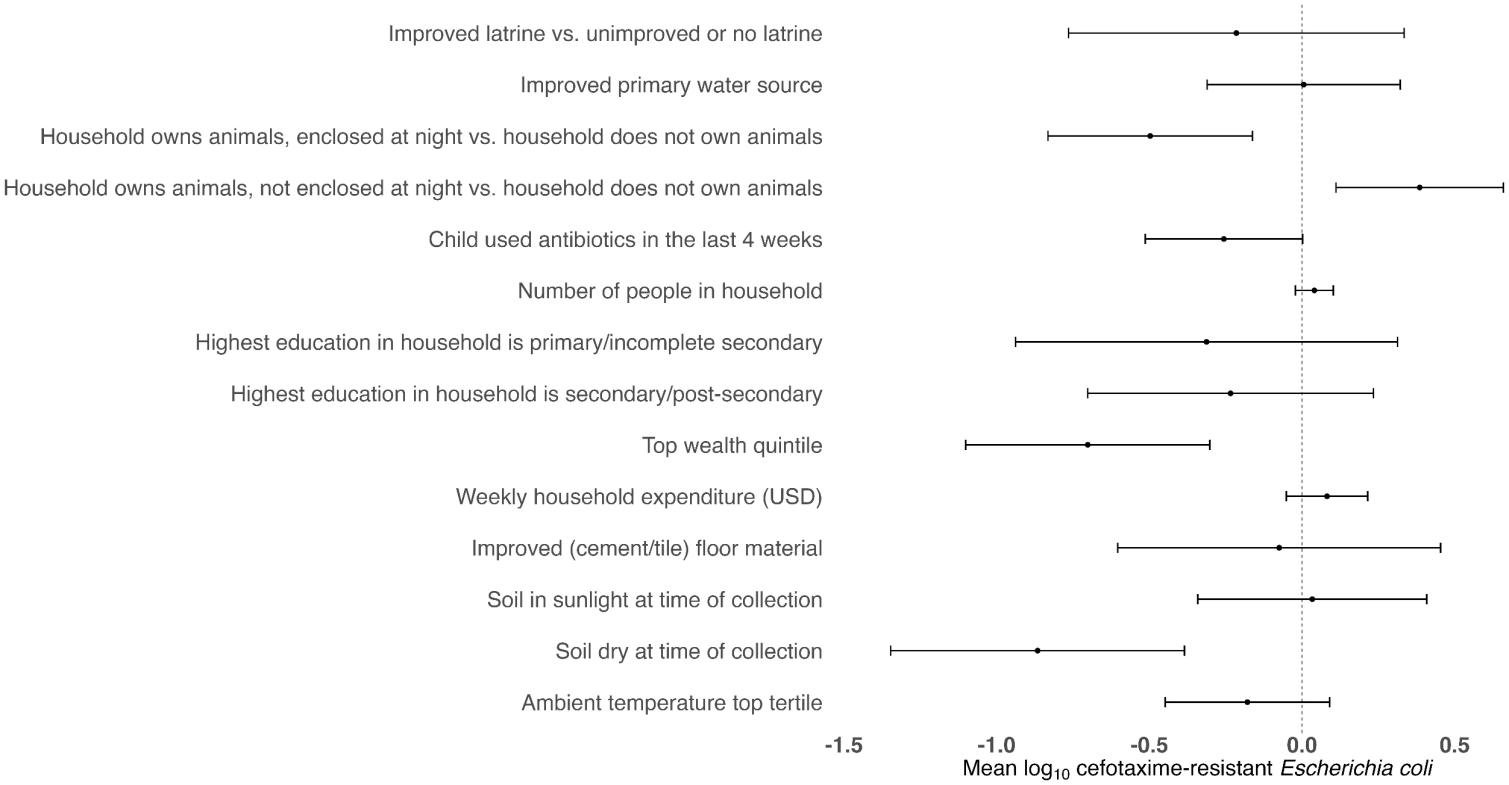
Forest plot of differences in mean log_10_-transformed most probable number (MPN) of cefotaxime-resistant *Escherichia coli* counts per dry gram of yard soil associated with household-level indicators of sanitation, animal ownership and management, child health, and environmental variables in multivariable models. Models include variables that were associated with the outcome with a p-value of <0.20 in bivariate analyses and also control for improved primary water source, highest education in the household, asset-based household wealth quintile, weekly household expenditure, number of people in the household and improved floor material. Circles indicate point estimates and horizontal lines indicate 95% confidence intervals.

In multivariable models that adjusted for potential confounders, animal cohabitation intensity had a strong association with the concentration of cefotaxime-resistant *E. coli* in yard soil. Specifically, compared to households that did not own animals, households that owned animals and kept them enclosed at night had a significantly lower concentration of cefotaxime-resistant *E. coli* in yard soil (Δlog_10_: -0.50 (-0.83, -0.16); p-value<0.005), whereas households that owned animals and did not enclose them at night had a higher concentration (Δlog_10_: 0.39 (0.11, 0.66); p-value<0.05) (Fig 2 and S3 Table). Households also had a significantly lower concentration of cefotaxime-resistant *E. coli* in yard soil if the soil was dry at the time of collection (Δlog_10_: -0.87 (-1.35, -0.39); p-value<0.005) and if the household was in the top wealth quintile (Δlog_10_: -0.70 (-1.10, -0.30); p-value<0.005) (Fig 2 and S3 Table). Any child in the household using antibiotics in the last four weeks was borderline associated with a lower concentration of cefotaxime-resistant *E. coli* in yard soil (Δlog_10_: -0.26 (-0.51, 0.0); p-value = 0.05) (Fig 2 and S3 Table). In a separate multivariable model that considered poultry ownership rather than overall animal ownership, owning poultry was not associated with cefotaxime-resistant *E. coli* concentration, while the soil being dry and greater household wealth remained associated and any child in the household using antibiotics in the last four weeks remained borderline associated with lower cefotaxime-resistant *E. coli* concentration (S4 Table). Sanitation and ambient temperature did not have an association with cefotaxime-resistant *E. coli* concentration in yard soil in multivariable models (Fig 2 and S3 and S4 Tables).

Findings for cefotaxime-resistant *E. coli* prevalence in yard soil were broadly similar to those for cefotaxime-resistant *E. coli* concentration. In bivariate models, households had significantly lower prevalence of cefotaxime-resistant *E. coli* if animals were enclosed at night, any child in the household used antibiotics in the last four weeks, if the soil was in sunlight or dry at time of collection, or if the ambient temperature was in the top tertile (S5 Table). While not statistically significant, households appeared to have lower prevalence of cefotaxime-resistant *E. coli* if they had a flush/pour flush latrine or the latrine was used by a single household (S5 Table). In contrast, while not significant, households appeared to have higher prevalence of cefotaxime-resistant *E. coli* if the household owned poultry, if children openly defecated, or if the ambient humidity was in the top tertile (S5 Table).

In multivariable models, lower prevalence of cefotaxime-resistant *E. coli* in yard soil was associated with any child in the household using antibiotics in the last four weeks (PR: 0.84 (0.70, 0.99); p-value = 0.04), soil being dry at time of collection (PR: 0.36 (0.26, 0.49); p-value<0.0005), ambient humidity in the top tertile (PR: 0.84 (0.72, 0.97); p-value = 0.02), and the household being in higher wealth quintiles (PR: 0.64 (0.46, 0.89); p-value = 0.01) (S6 and S7 Tables).

## Discussion

In our study in a peri-urban setting in Malawi, approximately two thirds of enrolled households with a child <5 years had cefotaxime-resistant *E. coli* in yard soil. Malawi is a low-income country and experiences high rates of AMR colonization both among humans and animals, consistent with prior evidence from other low-income countries [64–67]. A previous study in southern Malawi detected ESBL-producing *E. coli* or *Klebsiella pneumoniae* in 30% of animal feces (n = 290 samples) and 42% of human stools (n = 1190 samples) [68]. Strengthening AMR surveillance and regulations has been identified as a priority [69], and it is critical to investigate local drivers of AMR transmission. In our study, unenclosed domestic animals were associated with higher concentration of cefotaxime-resistant *E. coli* in yard soil. Enclosed domestic animals, soil being dry and higher household wealth were associated with lower concentration, and any child in the household using antibiotics in the last four weeks was borderline associated with lower concentration of cefotaxime-resistant *E. coli* in yard soil. Household sanitation was not associated with the prevalence or concentration of cefotaxime-resistant *E. coli* in yard soil.

Previous studies have identified soil as an important reservoir for AMR [23,30,31,70] and reported a range of prevalence values for antimicrobial-resistant *E. coli* in domestic, environmental and agricultural soils, including 14% of soil samples from a river basin in Tanzania [33], 42% of yard soil samples in Bangladesh [30], 68% of farm soil samples in Nigeria [32] and 89% of indoor soil floors in Bangladesh [29]. In Malawi, 77% of farm soil samples positive for *E. coli* showed cefotaxime resistance [71], and ESBL-producing bacteria (*E. coli, K. pneumoniae*) were found in 60% of soil samples from play and waste deposition areas [70]. Our finding of 69% prevalence in yard soil among peri-urban Malawian households is consistent with prior evidence. Recent studies in Malawi have also detected AMR in other environmental matrices such as farmland and household soil, vegetables, drains, standing water, and household drinking water [54,70,71]. However, in additional data from our study, the prevalence of cefotaxime-resistant *E. coli* in drinking water (8.4%) [54] was substantially lower than what we detected in yard soil, pointing to soil as a notable reservoir. Children <5 years in our study were reported to play, eat, sleep and crawl on the ground outside, presenting opportunities for soil exposure. Given previous evidence on the contribution of direct and indirect soil ingestion to child exposure to fecal contamination [39], our findings suggest that domestic soils can be a source of AMR exposure for young children.

As environmental pathways are increasingly recognized to facilitate AMR transmission, understanding drivers of AROs in environmental matrices is critical. Here, animal cohabitation, specifically nighttime enclosure of domestic animals, had a strong association with cefotaxime-resistant *E. coli* concentration in yard soil. Domestic animals often share living space with household members in low-income countries [29,72,73]. Animal presence has previously been associated with soil contamination with fecal indicators and AROs [29,74], and living in close proximity to animals has been shown to increase the odds of being colonized with AROs [75]. However, as 3.5 million people in Malawi experience chronic food insecurity [76], animal-source foods are critical for early childhood development [77]. While meat is infrequently sourced due to expense [77,78], the consumption of fish and eggs is common in Malawi [77]. Therefore, while owning domestic animals can present AMR risks, it can also support nutrition for household members, especially children.

There is also growing evidence on the contribution of specific animal management practices to soil contamination with AROs. Our findings align with a recent study in rural Bangladesh, where households who kept their animals inside the home at night or let them roam free inside the home or in the compound had higher prevalence and concentration of cefotaxime-resistant *E. coli* on indoor soil floors [29]. This could be due to antibiotic use in backyard animal husbandry and resulting dissemination of AMR via fecal waste from unenclosed or freely roaming domestic animals. A study in Kenya found that 80% of survey respondents utilized antibiotics in domestic livestock practices [12], while in our study 28% of households that owned animals gave them antibiotics (primarily to chickens). A recent study examining antimicrobial usage polices in Malawi found that animal husbandry is an area where further policies are needed, as clinically-relevant antimicrobials are not regulated for animal usage, and animal feces disposal is unregulated [10]. However, we found no association between animal antibiotic use and cefotaxime-resistant *E. coli* in yard soil. We did not record the specific antibiotics given to animals; it is possible that the antibiotics used for animals were not beta-lactams (e.g., tetracycline) and do not trigger ESBL production and/or cefotaxime resistance. Nonetheless, while antibiotic stewardship for domestic animals is recognized as an AMR control strategy [79], our findings highlight animal enclosure practices as an additional One Health approach to addressing AMR.

Poultry ownership was specifically associated with elevated cefotaxime-resistant *E. coli* in bivariate models in our study while this association was attenuated after controlling for potential confounders. Our findings are consistent with a 2017 study in Bangladesh that demonstrated domestic animals, especially chickens, contribute to fecal contamination in soil [34], and with a recent study that found associations between the number of chickens owned and cefotaxime-resistant *E. coli* on indoor soil floors [29]. In a study in Burkina Faso, children in households that owned poultry had greater odds of tetracycline resistance genes present in the gut compared to households that did not own poultry [80]. The specific contribution of poultry to fecal and AMR contamination in the domestic environment may be due to the difficulty in maintaining poultry enclosures [81]. Backyard poultry owners in low-income countries often prefer not to use enclosures because enclosed poultry require feed while free-range poultry can forage for food. Backyard poultry are also more likely to be allowed to roam freely inside living areas than larger animals, which are often tied up or kept in dedicated areas; roaming birds can contaminate the domestic environment as they scratch soil, forage and defecate. On the other hand, a study in Peru found that chicken corrals require an intensive process for maintaining hygiene [82], and corralling chickens increased *Campylobacter* infections among young children, compared to letting them range free [83]. Our survey collected enclosure information broadly for all animals in the compound and did not record whether poultry specifically were confined in an enclosure. However, among households that used nighttime enclosures, 50% owned poultry while 42% owned poultry and at least one other type of animal. Analyzing enclosure types and practices that households use specifically for poultry may provide further insight on how poultry management may affect cefotaxime-resistant *E. coli* contamination in yard soil.

Vulnerable water and sanitation infrastructure has been identified as a key barrier in addressing AMR in Malawi, as weak infrastructure perpetuates fecal-oral transmission and AMR dissemination [20,84]. In our study, cefotaxime-resistant *E. coli* concentration in soil was higher among households that had an unimproved latrine shared by a higher number of users, and in unadjusted regression models, multiple indicators of higher-quality sanitation, such as having an improved latrine or latrine used by a single household, were borderline associated with lower concentration of cefotaxime-resistant *E. coli* in yard soil. However, these associations were attenuated after controlling for potential confounders. Similarly, existing evidence on the association between sanitation and AMR is mixed and vulnerable to confounding by wealth. In a global analysis using human gut metagenome data (i.e., genetic material within the human gut) from 26 countries in Africa, North America, Europe, South America, and Asia, access to sanitation infrastructure that effectively isolates waste from human contact was associated with lower concentration of ARGs in the gut metagenome [14]. A 2018 modelling study found that lacking WaSH infrastructure was associated with higher AMR colonization in humans [19]. A study in Malawi found that having inadequate sanitation facilities was associated with higher AMR levels in drains and soil [70]. However, much of this evidence comes from observational studies, and it is possible that the findings are confounded by wealth (i.e., lower AMR in settings with better sanitation reflecting overall better health from higher wealth). In a randomized controlled trial in Bangladesh, on-site sanitation improvements did not decrease fecal contamination of courtyard soil [42], and there is mixed evidence on how effectively on-site sanitation systems isolate fecal waste from the environment in low-income countries [85]. Taken together with this prior evidence, our findings suggest that household-level sanitation practices may not fully capture drivers of cefotaxime resistance in environmental compartments such as soil; integrating other variables, such as community-level infrastructure and practices, environmental factors and animal husbandry, may identify critical additional drivers [86]. Future randomized sanitation trials that measure AMR in the environment or among residents can help generate unconfounded evidence on the relationship between sanitation status and AMR.

In our analysis, child health variables such as diarrheal and respiratory infections, which are common drivers of pediatric antibiotic use, were not associated with cefotaxime-resistant *E. coli* in yard soil. In bivariate models, child antibiotic use in the last month was associated with lower cefotaxime-resistant *E. coli* prevalence and borderline associated with lower cefotaxime-resistant *E. coli* concentration in soil. In multivariable models, child antibiotic use remained borderline associated with lower concentration and significantly associated with lower prevalence of cefotaxime-resistant *E. coli* in yard soil. Notably, approximately a third of children in our study used antibiotics in the last month. The most commonly reported antibiotics were amoxicillin and trimethoprim/sulfamethoxazole. While trimethoprim/sulfamethoxazole belong to the sulfonamide class of antibiotics [87], amoxicillin belongs to the penicillin class and is a beta-lactam antibiotic like cefotaxime [88]. Therefore, frequent amoxicillin use by children would be expected to be associated with ESBL-production and/or cefotaxime resistance in children’s guts and consequently in domestic soils, given that 25% of children in our study open defecated within the compound. Our finding of borderline association between child antibiotic use and lower cefotaxime resistant *E. coli* in yard soil conflicts with prior research that suggests that environmental AMR may result from high antibiotic use through the excretion of antibiotic residues [89], while it also supports existing evidence that environmental transmission may contribute more to human colonization with AMR than the direct consumption of antimicrobials [19]. We note that self-reported antibiotic use can be inaccurate. One study assessing antibiotics sold in Malawi found high numbers of unregistered antibiotics and inconsistent dosage between pills [90], while another study in Malawi that analyzed 56 antibiotic samples sold by drug retailers found six sub-standard (i.e., does not meet drug standards) and one falsified (i.e., distorts the drug’s true identity) antibiotics/antimalarials [91]. We therefore asked to see the reported antibiotic administered to children. While we were able to confirm the reported antibiotic only for a small subset of children whose caregivers had the packaging available, in more than 75% of these instances, the packaging matched the reported antibiotic. Similarly, a previous study in eight low- and middle-income countries found that caregiver-reported pediatric antibiotic use matched the children’s medical records [92]. Our findings are consistent with prior studies showing high antibiotic use among young children in sub-Saharan Africa. In a recent study in Kenya, caregivers reported that 63% of children <5 years had taken antibiotics in the previous 90 days; caregivers were asked to name the specific antibiotic used, and data were excluded if the named medication was not an antibiotic [93]. While we did not record antibiotic consumption by household members other than children <5 years, our findings suggest that factors outside of human consumption of antibiotics may be dominant drivers of cefotaxime resistance in the home environment in peri-urban Malawi.

Some environmental factors, such as soil being dry, were associated with lower cefotaxime-resistant *E. coli* concentration in yard soil in our study. In contrast, while high ambient temperature was associated with lower cefotaxime-resistant *E. coli* concentration in bivariate models, this relationship was attenuated in multivariable models, and ambient humidity was not associated with cefotaxime-resistant *E. coli* concentration in bivariate or multivariate models. Our findings are consistent with previous evidence on the effect of moisture on *E. coli* and antimicrobial behavior. *E. coli* proliferates in soil under favorable environmental conditions, including higher moisture content [30,94]. Sunlight can inactivate microorganisms [95] and may break down antibiotic residues in soil; when exposed to sunlight, oxytetracycline and tetracycline, common broad-spectrum antibiotics used in humans and animals [96], lose antibiotic potential [97] and therefore may not trigger resistance. On the other hand, our findings differ from previous evidence on the relationship between ambient temperature/humidity and AMR. In a global study utilizing AMR databases, AMR colonization in inpatients was more common in areas with warmer temperatures [19]. An ecological study in China utilizing human clinical data found that, although ambient humidity confounds with ambient temperature, the median relative humidity of the area may influence the proliferation of AMR alongside ambient temperatures [62]. Our findings of no association with these weather parameters may reflect our data collection period during the cool, dry winter season in Malawi. In contrast, associations between ambient temperature and AMR in human clinical data have been derived from multi-year simulation studies, which better capture temperature variations [98]. We also did not analyze soil temperature specifically, which may influence the survival and proliferation of ARGs in soil [99]. Understanding how weather affects AROs is critical, given increasing temperatures and rainfall intensity in many world regions where AMR is common.

Our study had some limitations. First, we solely measured cefotaxime-resistant *E. coli*, which may not capture other AMR classes or specific resistant pathogens. Cefotaxime is a third generation cephalosporin and a broad-spectrum antibiotic [52,100]. Cefotaxime resistance is conferred by ESBL production; ESBL-producing organisms have multi-drug resistance against penicillins and cephalosporins, including cefotaxime [101]. Therefore, our study used a validated protocol [57] to quantify cefotaxime-resistant *E. coli* in yard soil as a proxy for ESBL-producing *E. coli* [52], which is widely monitored as a sentinel organism for AMR in environmental matrices, such as in the World Health Organization’s Tricycle global AMR surveillance protocol [53]. We also did not measure cefotaxime-resistant *E. coli* or other AROs in human or animal stool to corroborate the associations with sanitation and animal management practices. Additionally, our cross-sectional data collection only captured the dry/cold season in Malawi; the concentration of cefotaxime-resistant *E. coli* in yard soil and factors associated with it may differ during warm/rainy seasons. Further, due to our observational cross-sectional study design, we could only establish association, not causation, between household and environmental variables and cefotaxime-resistant *E. coli* in yard soil. While we accounted for a range of potential confounders (water source, education, socio-demographics), the possibility of confounding remains. Finally, we collected self-reported data on household practices using a structured questionnaire. Respondents may remember events and answer survey questions inaccurately. However, we have no reason to expect that any such misreporting would be differential with respect to our study exposure and outcomes variables; any non-differential misreporting would be expected to attenuate the observed associations.

## Conclusion

Findings from this study suggest that while sanitation and child health did not influence cefotaxime-resistant *E. coli* in yard soil, environmental and animal husbandry characteristics were significantly associated and child antibiotic use was borderline associated with the concentration and/or prevalence of cefotaxime-resistant *E. coli* in this peri-urban study setting. Results highlight the importance of taking a One Health approach to incorporate factors related to animals and the environment in global AMR mitigation strategies. Future studies should investigate the influence of specific animal husbandry practices, including animal cohabitation intensity and animal types, on cefotaxime-resistant *E. coli* in yard soil, other matrices in the domestic environment and among household members.

## Supporting information

S1 Checklist *PLOS Global Public Health* inclusivity in global research questionnaire. (DOCX)

S1 Table Socio-demographics and water, sanitation and hygiene conditions of enrolled households. (DOCX)

S1 Fig Moisture content (%) of soil, stratified by enumerator observation if the soil was wet/dry at time of collection. The X-axis reflects whether the soil was wet or dry at time of collection, and the Y-axis reflects the average moisture content (%) of the sample. The bottommost and topmost horizontal lines represent the lower and upper limits (excluding outliers), the bottom and top borders of the box represent the first and third quartiles, and the bold horizontal line represents the median of moisture content (%).
(TIF)

S2 Fig Mean log_10_-transformed most probable number (MPN) of cefotaxime-resistant *Escherichia coli* among cross-categories of animal ownership and husbandry characteristics. Y-axis variables include animals the household or compound owns, including any animals, dogs/cats, and poultry. X-axis variables include specific animal husbandry characteristics, including if animals are enclosed in the daytime/nighttime, household administers antibiotics to animals, and animal feces observed in the compound during sampling. The heatmap gradient reflects the mean log10-transformed MPN of cefotaxime-resistant *E. coli* in yard soil, where darker colors represent higher MPN values. Boundaries indicate complementary variables.
(TIF)

S2 Table Bivariate associations between household environmental characteristics and concentration of cefotaxime-resistant *E. coli* in yard soil. Bolded values indicate associations with p-value <0.20.
(DOCX)

S3 Table Adjusted associations between household environmental characteristics, animal ownership and concentration of cefotaxime-resistant *E. coli* in yard soil. Models include variables that were associated with the outcome with a p-value of <0.20 in bivariate analyses and also control for household’s primary water source, education, socioeconomics, household size, and indoor floor material. Bolded values indicate associations with p-value <0.05.
(DOCX)

S4 Table Adjusted associations between household environmental characteristics, poultry ownership and concentration of cefotaxime-resistant *E. coli* in yard soil. Models include variables that were associated with the outcome with a p-value of <0.20 in bivariate analyses and also control for household’s primary water source, education, socioeconomics, household size, and indoor floor material. Bolded values indicate associations with p-value <0.05.
(DOCX)

S5 Table Bivariate associations between household environmental characteristics and prevalence of cefotaxime-resistant *E. coli* in yard soil. Bolded values indicate associations with p-value <0.05.
(DOCX)

S6 Table Adjusted associations between household environmental characteristics, animal ownership and prevalence of cefotaxime-resistant *E. coli* in yard soil. Models include variables that were associated with the outcome with a p-value of <0.20 in bivariate analyses and also control for household’s primary water source, education, socioeconomics, household size, and indoor floor material. Bolded values indicate associations with p-value <0.05.
(DOCX)

S7 Table Adjusted associations between household environmental characteristics, poultry ownership and prevalence of cefotaxime-resistant *E. coli* in yard soil. Models include variables that were associated with the outcome with a p-value of <0.20 in bivariate analyses and also control for household’s primary water source, education, socioeconomics, household size, and indoor floor material. Bolded values indicate associations with p-value <0.05.
(DOCX)
