# Supplementary material for: Animal husbandry and environmental conditions are associated with cefotaxime-resistant Escherichia coli in yard soil in peri-urban Malawi
Source: PLOS Glob Public Health. 2026 Jul 13;6(7):e0006264. doi: 10.1371/journal.pgph.0006264 (PMC13362151; doi:10.1371/journal.pgph.0006264)
Supplement: S1 Table — (DOCX) [file pgph.0006264.s002.docx]

**S1 Table. Socio-demographics and water, sanitation and hygiene conditions of enrolled households**

| Socio-demographics | N=237 |
| --- | --- |
| Number of individuals living in household, mean (SD) | 5.3 (2.0) |
| Children <5 years | 1.2 (0.5) |
| Children 5-16 years | 1.4 (1.2) |
| Adults >16 years | 2.7 (1.3) |
| Respondent’s age, mean (SD) | 31.1 (10.2) |
| Respondent can read and write, % (n) | 86.5 (205) |
| Respondent’s education, % (n) |  |
| None | 1.7 (4) |
| Primary (partial/complete) | 42.6 (101) |
| Secondary (partial/complete) | 49.8 (118) |
| Post-secondary | 5.9 (14) |
| Highest education in the household, % (n) |  |
| None | 0.4 (1) |
| Primary (partial/complete) | 17.3 (41) |
| Secondary (partial/complete) | 62.9 (149) |
| Post-secondary | 18.1 (43) |
| Weekly household expenditure (USD), mean (SD) | 17.0 (15.5) |
| Household had electricity in the last 7 days, % (n) | 69.2 (164) |
| Household owns, % (n) |  |
| Refrigerator | 23.6 (56) |
| Mosquito net | 70.0 (166) |
| Mobile phone | 86.5 (205) |
| Water, sanitation and hygiene | N=237 |
| Improved primary water source, % (n) | 84.4 (200) |
| Primary water source, % (n) |  |
| Tubewell/borehole | 21.5 (51) |
| Protected spring | 0.4 (1) |
| Unprotected dug well | 0.8 (2) |
| Protected dug well (has concrete lining) | 5.9 (14) |
| Surface water (river, dam, lake, pond, stream, canal, channel) | 0.8 (2) |
| Piped water into dwelling | 8.4 (20) |
| Piped water into yard/plot | 19.0 (45) |
| Piped water outside the compound | 29.1 (69) |
| Vendor water (kiosk) | 12.7 (30) |
| Other | 1.3 (3) |
| Improved latrine, % (n) | 19.4 (46) |
| Type of latrine, % (n)^a^ |  |
| No latrine | 1.3 (3) |
| Flush/pour flush | 1.3 (3) |
| Flush to piped sewer | 0.4 (1) |
| Flush to septic tank | 7.2 (17) |
| Flush to pit latrine | 0.8 (2) |
| Dry pit latrine | 0.4 (1) |
| Pit latrine with slab | 65.0 (154) |
| Pit latrine without slab/open pit | 27.8 (66) |
| Twin pit with slab | 0.4 (1) |
| Twin pit without slab | 0.4 (1) |
| Other | 1.3 (3) |
| Latrine shared with other households, % (n) | 70.9 (168) |
| Number of people using the latrine, mean (SD) | 11.0 (8.3) |
| Children <5 years defecate in, % (n)^a^ |  |
| Latrine | 40.1 (95) |
| Potty | 8.0 (19) |
| Nappy/diaper | 38.8 (92) |
| Open defecate in compound | 24.9 (59) |
| Open defecate outside of compound | 4.6 (11) |
| Child feces disposed of, % (n)^a^ |  |
| Child used toilet/latrine | 29.5 (70) |
| Put/rinsed into toilet or latrine | 64.6 (153) |
| Put/rinsed into drain or ditch | 0.8 (2) |
| Thrown into garbage (solid waste) | 0.8 (2) |
| Buried | 0.4 (1) |
| Other | 10.1 (24) |
| Feces (human/animal) observed within 2x2 meters of soil sampling area, % (n) | 8.4 (20) |
| Household has handwashing station with water and soap, % (n) | 4.6 (11) |
| Respondent reports washing hands, % (n) ^a^ | N = 237 |
| After defecation | 91.6 (217) |
| After handling child’s waste | 62.4 (148) |
| After handling domestic animals | 8.0 (19) |
| After handling animal feces | 7.2 (17) |
| After working (garden, market, etc) | 21.9 (52) |
| After eating | 80.6 (191) |
| Before eating | 92.8 (220) |
| Before preparing food | 67.1 (159) |
| Before feeding child | 41.8 (99) |
| Before handling water (storage) | 24.1 (57) |
| Other | 3.8 (9) |

SD: Standard deviation.

^a^ Respondent could select multiple answers.
