# Supplementary material for: Animal husbandry and environmental conditions are associated with cefotaxime-resistant Escherichia coli in yard soil in peri-urban Malawi
Source: PLOS Glob Public Health. 2026 Jul 13;6(7):e0006264. doi: 10.1371/journal.pgph.0006264 (PMC13362151; doi:10.1371/journal.pgph.0006264)
Supplement: S2 Table — Bolded values indicate associations with p-value <0.20. (DOCX) [file pgph.0006264.s005.docx]

**S2 Table. Bivariate associations between household environmental characteristics and concentration of cefotaxime-resistant *E. coli* in yard soil.** Bolded values indicate associations with p-value <0.20.

|  | Yes | | No | |  |  |
| --- | --- | --- | --- | --- | --- | --- |
|  | N | log_10_-MPN  Mean (SD) | N | log_10_-MPN  Mean (SD) | $\Delta$log_10_-MPN  (95% CI) | p-value |
| Sanitation |  |  |  |  |  |  |
| Improved latrine | 45 | 0.60 (1.09) | 186 | 0.98 (1.13) | **-0.38 [-0.81, 0.06]** | **0.09** |
| Flush/pour flush latrine | 20 | 0.66 (1.40) | 211 | 0.93 (1.10) | -0.27 [-0.83, 0.30] | 0.35 |
| Latrine used by single household | 65 | 0.60 (1.05) | 163 | 1.02 (1.12) | **-0.42 [-0.72, -0.12]** | **0.01** |
| Children in household openly defecate | 68 | 1.02 (1.19) | 163 | 0.85 (1.10) | 0.17 [-0.24, 0.58] | 0.41 |
| Feces observed within 2x2 m of soil sampling area | 20 | 0.89 (1.21) | 211 | 0.91 (1.12) | -0.02 [-0.65, 0.62] | 0.96 |
| Animal ownership and management |  |  |  |  |  |  |
| Household owns |  |  |  |  |  |  |
| Animals | 56 | 1.04 (1.14) | 175 | 0.86 (1.12) | 0.18 [-0.10, 0.47] | 0.21 |
| Poultry | 36 | 1.04 (1.08) | 195 | 0.88 (1.14) | **0.16 [-0.08, 0.39]** | **0.19** |
| Dogs/cats | 30 | 0.85 (1.18) | 201 | 0.91 (1.12) | -0.06 [-0.51, 0.38] | 0.78 |
| Compound keeps |  |  |  |  |  |  |
| Animals | 175 | 0.92 (1.13) | 56 | 0.86 (1.11) | 0.06 [-0.31, 0.42] | 0.76 |
| Poultry | 143 | 0.92 (1.13) | 88 | 0.88 (1.13) | 0.04 [-0.19, 0.28] | 0.72 |
| Dogs/cats | 109 | 0.84 (1.13) | 122 | 0.96 (1.12) | -0.13 [-0.45, 0.20] | 0.45 |
| Animals observed within 2x2m of soil sampling area | 16 | 0.97 (1.14) | 215 | 0.90 (1.13) | 0.07 [-0.49, 0.63] | 0.82 |
| Animal feces observed in compound | 20 | 0.89 (1.21) | 211 | 0.91 (1.12) | -0.02 [-0.65, 0.62] | 0.96 |
| Animals enclosed during day | 7 | 0.66 (0.77) | 49 | 1.10 (1.18) | -0.43 [-1.13, 0.27] | 0.23 |
| Animals enclosed at night | 12 | 0.16 (0.55) | 44 | 1.28 (1.15) | **-1.12 [-1.56, -0.67]** | **<0.0005** |
| Household gave antibiotics in last 4 weeks to: |  |  |  |  |  |  |
| Animals | 16 | 0.83 (1.27) | 40 | 1.13 (1.09) | -0.30 [-0.98, 0.38] | 0.38 |
| Poultry | 8 | 0.87 (1.21) | 48 | 1.07 (1.14) | -0.20 [-1.11, 0.70] | 0.66 |
| Dogs/cats | 8 | 0.79 (1.40) | 48 | 1.09 (1.10) | -0.30 [-1.40, 0.81] | 0.60 |
| Child health |  |  |  |  |  |  |
| Any child in household had in last 7 days: |  |  |  |  |  |  |
| Diarrhea | 47 | 0.94 (1.17) | 184 | 0.90 (1.12) | 0.05 [-0.36, 0.45] | 0.83 |
| Acute respiratory infection | 117 | 0.86 (1.13) | 114 | 0.95 (1.13) | -0.09 [-0.43, 0.24] | 0.60 |
| Acute respiratory infection with fever | 63 | 0.76 (1.13) | 168 | 0.96 (1.12) | -0.20 [-0.58, 0.19] | 0.31 |
| Fever | 86 | 0.80 (1.17) | 145 | 0.97 (1.10) | -0.16 [-0.44, 0.11] | 0.23 |
| Any child in household used antibiotics in last 4 weeks | 85 | 0.77 (1.15) | 146 | **0.98 (1.11)** | **-0.21 [-0.51, 0.09]** | **0.17** |
| Environmental factors |  |  |  |  |  |  |
| Soil sampling area in sunlight at time of collection | 90 | 0.74 (1.19) | 141 | 1.01 (1.08) | **-0.27 [-0.64, 0.11]** | **0.16** |
| Soil sampling area dry at time of collection | 53 | 0.19 (0.92) | 178 | 1.12 (1.10) | **-0.92 [-1.29, -0.56]** | **<0.0005** |
| Ambient temperature in top tertile | 58 | 0.67 (1.07) | 173 | 0.98 (1.14) | **-0.31 [-0.59, -0.03]** | **0.03** |
| Ambient humidity in top tertile | 109 | 0.98 (1.06) | 122 | 0.84 (1.18) | 0.14 [-0.26, 0.53] | 0.50 |

Log_10_-MPN: Log_10_transformed most-probable number cefotaxime-resistant *E. coli*. ($\Delta$log_10_-MPN: difference between binary log_10_ transformed most-probable number cefotaxime-resistant *E. coli*; SD: Standard Deviation; CI: Confidence Interval.
