# Supplementary material for: Animal husbandry and environmental conditions are associated with cefotaxime-resistant Escherichia coli in yard soil in peri-urban Malawi
Source: PLOS Glob Public Health. 2026 Jul 13;6(7):e0006264. doi: 10.1371/journal.pgph.0006264 (PMC13362151; doi:10.1371/journal.pgph.0006264)
Supplement: S3 Table — Models include variables that were associated with the outcome with a p-value of <0.20 in bivariate analyses and also control for household’s primary water source, education, socioeconomics, household size, and indoor floor material. Bolded values indicate associations with p-value <0.05. (DOCX) [file pgph.0006264.s006.docx]

**S3 Table.** **Adjusted associations between household environmental characteristics, animal ownership and concentration of cefotaxime-resistant *E. coli* in yard soil.** Models include variables that were associated with the outcome with a p-value of <0.20 in bivariate analyses and also control for household’s primary water source, education, socioeconomics, household size, and indoor floor material. Bolded values indicate associations with p-value <0.05.

| Variable | Δlog_10_-MPN/dry gram (95% CI) | p-value |
| --- | --- | --- |
| Improved latrine (vs. unimproved or no latrine) | -0.22 [-0.76, 0.33] | 0.44 |
| Household owns animals, enclosed at night (vs. no animals owned) | **-0.50 [-0.83, -0.16]** | **<0.005** |
| Household owns animals, not enclosed at night (vs. no animals owned) | **0.39 [0.11, 0.66]** | **<0.05** |
| Child used antibiotics in the last 4 weeks | **-0.26 [-0.51, 0.0]** | **0.05** |
| Soil in sunlight at time of collection | 0.03 [-0.34, 0.41] | 0.86 |
| Soil dry at time of collection | **-0.87 [-1.35, -0.39]** | **<0.005** |
| Ambient temperature in top tertile | -0.18 [-0.45, 0.09] | 0.19 |
| Improved primary water source | 0.01 [-0.31, 0.32] | 0.97 |
| Highest education in household is primary/incomplete secondary (vs. no formal education) | -0.31 [-0.94, 0.31] | 0.33 |
| Highest education in household is secondary/post-secondary (vs. no formal education) | -0.23 [-0.70, 0.23] | 0.33 |
| 2^nd^ wealth quintile (vs. bottom quintile) ^a^ | -0.23 [-0.58, 0.12] | 0.20 |
| 3^rd^ wealth quintile (vs. bottom quintile) ^a^ | -0.09 [-0.56, 0.39] | 0.72 |
| 4^th^ wealth quintile (vs. bottom quintile) ^a^ | -0.22 [-0.66, 0.22] | 0.33 |
| 5^th^ wealth quintile (vs. bottom quintile) ^a^ | **-0.70 [-1.10, -0.30]** | **<0.005** |
| Weekly household expenditure (USD) ^b^ | 0.08 [-0.05, 0.22] | 0.23 |
| Number of people in household | 0.04 [-0.02, 0.10] | 0.20 |
| Improved (cement/tile) floor material | -0.07 [-0.60, 0.45] | 0.78 |

Δlog_10_-MPN: difference between binary log_10_ transformed most-probable number cefotaxime-resistant *E. coli*; CI: Confidence Interval

^a^ Wealth quintile determined by principal component analysis of assets owned by the household. The quintiles range from poorest (1) to wealthiest (5).

^b^ Associations reported per $10 USD increase in weekly household expenditure.

^d^ Asked among the 24 children whose antibiotic was ineffective
