# Supplementary material for: Animal husbandry and environmental conditions are associated with cefotaxime-resistant Escherichia coli in yard soil in peri-urban Malawi
Source: PLOS Glob Public Health. 2026 Jul 13;6(7):e0006264. doi: 10.1371/journal.pgph.0006264 (PMC13362151; doi:10.1371/journal.pgph.0006264)
Supplement: S4 Table — Models include variables that were associated with the outcome with a p-value of <0.20 in bivariate analyses and also control for household’s primary water source, education, socioeconomics, household size, and indoor floor material. Bolded values indicate associations with p-value <0.05. (DOCX) [file pgph.0006264.s007.docx]

**S4 Table. Adjusted associations between household environmental characteristics, poultry ownership and concentration of cefotaxime-resistant *E. coli* in yard soil.** Models include variables that were associated with the outcome with a p-value of <0.20 in bivariate analyses and also control for household’s primary water source, education, socioeconomics, household size, and indoor floor material. Bolded values indicate associations with p-value <0.05.

| Variable | Δlog_10_-MPN/dry gram [95% CI] | p-value |
| --- | --- | --- |
| Improved latrine (vs. unimproved or no latrine) | -0.30 [-0.88, 0.29] | 0.32 |
| Household owns poultry | 0.15 [-0.15, 0.45] | 0.32 |
| Child used antibiotics in the last 4 weeks | **-0.25 [-0.50, 0.0]** | **0.05** |
| Soil in sunlight at time of collection | 0.12 [-0.24, 0.47] | 0.52 |
| Soil dry at time of collection | **-0.95 [-1.44, -0.46]** | **<0.005** |
| Ambient temperature in top tertile | -0.12 [-0.37, 0.13] | 0.35 |
| Improved primary water source | 0.03 [-0.29, 0.34] | 0.86 |
| Highest education in household is primary/incomplete secondary (vs. no formal education) | -0.26 [-0.91, 0.39] | 0.43 |
| Highest education in household is secondary/post-secondary (vs. no formal education) | -0.18 [-0.64, 0.29] | 0.46 |
| 2^nd^ Wealth quintile ^a^ | -0.18 [-0.54, 0.18] | 0.33 |
| 3^rd^ Wealth quintile ^a^ | -0.10 [-0.63, 0.43] | 0.71 |
| 4^th^ Wealth quintile ^a^ | -0.21 [-0.65, 0.22] | 0.34 |
| 5^th^ Wealth quintile ^a^ | **-0.75 [-1.15, -0.36]** | **<0.005** |
| Weekly household expenditure ^b^ | 0.09 [-0.04, 0.22] | 0.19 |
| Number of people in household | 0.04 [-0.02, 0.11] | 0.19 |
| Improved (cement/tile) floor material | -0.08 [-0.61, 0.45] | 0.77 |

Δlog_10_-MPN: difference between binary log_10_ transformed most-probable number cefotaxime-resistant *E. coli*; CI: Confidence Interval

^a^ Wealth quintile determined by assets owned by the household. The quintile ranges from poorest (1) to wealthiest (5).

^b^ Association reported per $10 USD spent.
