# Supplementary material for: Animal husbandry and environmental conditions are associated with cefotaxime-resistant Escherichia coli in yard soil in peri-urban Malawi
Source: PLOS Glob Public Health. 2026 Jul 13;6(7):e0006264. doi: 10.1371/journal.pgph.0006264 (PMC13362151; doi:10.1371/journal.pgph.0006264)
Supplement: S5 Table — Bolded values indicate associations with p-value <0.05. (DOCX) [file pgph.0006264.s008.docx]

**S5 Table. Bivariate associations between household environmental characteristics and prevalence of cefotaxime-resistant *E. coli* in yard soil.** Bolded values indicate associations with p-value <0.05.

|  | Yes | | No | |  |  |
| --- | --- | --- | --- | --- | --- | --- |
|  | N | Prevalence  % (n) | N | Prevalence  % (n) | Prevalence ratio  (95% CI) | p-value |
| Sanitation |  |  |  |  |  |  |
| Improved latrine | 45 | 64.4 (29) | 188 | 69.7 (131) | 0.93 [0.71, 1.21] | 0.56 |
| Flush/pour flush latrine | 20 | 50.0 (10) | 213 | 70.4 (150) | **0.71 [0.47, 1.08]** | **0.11** |
| Latrine used by single household | 65 | 63.1 (41) | 165 | 71.5 (118) | **0.88 [0.74, 1.05]** | **0.15** |
| Children in household openly defecate | 68 | 75.0 (51) | 165 | 66.1 (109) | **1.14 [0.94, 1.37]** | **0.19** |
| Feces observed within 2x2 m of soil sampling area | 20 | 75.0 (15) | 213 | 68.1 (145) | 1.10 [0.87, 1.39] | 0.41 |
| Animal ownership and management |  |  |  |  |  |  |
| Household owns |  |  |  |  |  |  |
| Animals | 56 | 73.2 (41) | 177 | 67.2 (119) | 1.09 [0.94, 1.27] | 0.27 |
| Poultry | 36 | 77.78 (28) | 197 | 67.0 (132) | **1.16 [0.96, 1.40]** | **0.11** |
| Dogs/cats | 30 | 60.0 (18) | 203 | 70.0 (142) | 0.86 [0.67, 1.09] | 0.22 |
| Compound keeps |  |  |  |  |  |  |
| Animals | 176 | 70.5 (124) | 57 | 63.2 (36) | 1.12 [0.87, 1.43] | 0.39 |
| Poultry | 144 | 70.1 (101) | 89 | 66.3 (59) | 1.06 [0.89, 1.26] | 0.52 |
| Dogs/cats | 110 | 65.5 (72) | 123 | 71.5 (88) | 0.92 [0.75, 1.12] | 0.39 |
| Animals observed within 2x2m of soil sampling area | 16 | 68.8 (11) | 217 | 68.7 (149) | 1.00 [0.81, 1.24] | 0.99 |
| Animal feces observed in compound | 20 | 75.0 (15) | 213 | 68.1 (145) | 1.10 [0.87, 1.39] | 0.41 |
| Animals enclosed during day | 7 | 85.7 (6) | 49 | 71.4 (35) | 1.20 [0.82, 1.76] | 0.35 |
| Animals enclosed at night | 12 | 50.0 (6) | 44 | 79.5 (35) | **0.63 [0.41, 0.95]** | **0.03** |
| Household gave antibiotics in last 4 weeks to: |  |  |  |  |  |  |
| Animals | 16 | 62.5 (10) | 40 | 77.5 (31) | 0.81 [0.52, 1.25] | 0.33 |
| Poultry | 8 | 75.0 (6) | 48 | 72.9 (35) | 1.03 [0.64, 1.65] | 0.91 |
| Dogs/cats | 8 | 50.0 (4) | 48 | 77.1 (37) | 0.65 [0.28, 1.50] | 0.31 |
| Child health |  |  |  |  |  |  |
| Any child in household had in last 7 days: |  |  |  |  |  |  |
| Diarrhea | 47 | 72.3 (34) | 186 | 67.7 (126) | 1.07 [0.84, 1.35] | 0.58 |
| Acute respiratory infection | 117 | 67.5 (79) | 116 | 69.8 (81) | 0.97 [0.79, 1.18] | 0.74 |
| Acute respiratory infection with fever | 63 | 65.1 (41) | 170 | 70.0 (119) | 0.93 [0.76, 1.14] | 0.49 |
| Fever | 86 | 64.0 (55) | 147 | 71.4 (105) | 0.90 [0.75, 1.06] | 0.21 |
| Any child in household used antibiotics in last 4 weeks | 85 | 61.2 (52) | 148 | 73.0 (108) | **0.84 [0.71, 0.99]** | **0.04** |
| Environmental factors |  |  |  |  |  |  |
| Soil sampling area in sunlight at time of collection | 90 | 56.7 (51) | 143 | 76.2 (109) | **0.74 [0.60, 0.92]** | **0.01** |
| Soil sampling area dry at time of collection | 53 | 28.3 (15) | 180 | 80.6 (145) | **0.35 [0.25, 0.49]** | **<0.0005** |
| Ambient temperature in top tertile | 58 | 58.6 (34) | 175 | 72.0 (126) | **0.81 [0.70, 0.94]** | **<0.05** |
| Ambient humidity in top tertile | 111 | 74.8 (83) | 122 | 63.1 (77) | **1.19 [0.97, 1.45]** | **0.10** |

Log10-MPN: log10-transformed most-probable number cefotaxime-resistant *E. coli*; Δlog10-MPN: difference between binary log10 transformed most-probable number cefotaxime-resistant *E. coli*; SD: Standard Deviation; CI: Confidence Interval
