# Supplementary material for: Animal husbandry and environmental conditions are associated with cefotaxime-resistant Escherichia coli in yard soil in peri-urban Malawi
Source: PLOS Glob Public Health. 2026 Jul 13;6(7):e0006264. doi: 10.1371/journal.pgph.0006264 (PMC13362151; doi:10.1371/journal.pgph.0006264)
Supplement: S6 Table — Models include variables that were associated with the outcome with a p-value of <0.20 in bivariate analyses and also control for household’s primary water source, education, socioeconomics, household size, and indoor floor material. Bolded values indicate associations with p-value <0.05. (DOCX) [file pgph.0006264.s009.docx]

**S6 Table. Adjusted associations between household environmental characteristics, animal ownership and prevalence of cefotaxime-resistant *E. coli* in yard soil.** Models include variables that were associated with the outcome with a p-value of <0.20 in bivariate analyses and also control for household’s primary water source, education, socioeconomics, household size, and indoor floor material. Bolded values indicate associations with p-value <0.05.

| Variable | Prevalence ratio (95% CI) | p-value |
| --- | --- | --- |
| Flush/pour flush latrine | 0.90 [0.68, 1.19] | 0.47 |
| Latrine used by single household | 0.97 [0.77, 1.24] | 0.83 |
| Children openly defecate | 1.15 [0.93, 1.41] | 0.20 |
| Household owns animals, enclosed at night (vs. no animals owned) | 0.83 [0.58, 1.19] | 0.32 |
| Household owns animals, not enclosed at night (vs. no animals owned) | 1.10 [0.95, 1.28] | 0.20 |
| Child used antibiotics in the last 4 weeks | **0.84 [0.70, 0.99]** | **0.04** |
| Soil in sunlight at time of collection | 0.95 [0.80, 1.12] | 0.54 |
| Soil dry at time of collection | **0.36 [0.26, 0.49]** | **<0.0005** |
| Ambient temperature in top tertile | 0.92 [0.71, 1.18] | 0.51 |
| Ambient humidity in top tertile | **0.84 [0.72, 0.97]** | **0.02** |
| Improved primary water source | 1.01 [0.84, 1.21] | 0.94 |
| Highest education in household is primary/incomplete secondary (vs. no formal education) | 0.88 [0.65, 1.19] | 0.40 |
| Highest education in household is secondary/post-secondary (vs. no formal education) | 0.93 [0.75, 1.16] | 0.55 |
| 2^nd^ Wealth quintile (vs. bottom quintile) ^a^ | 0.82 [0.65, 1.04] | 0.10 |
| 3^rd^ Wealth quintile (vs. bottom quintile) ^a^ | 0.82 [0.64, 1.04] | 0.10 |
| 4^th^ Wealth quintile (vs. bottom quintile) ^a^ | 0.81 [0.59, 1.11] | 0.20 |
| 5^th^ Wealth quintile (vs. bottom quintile) ^a^ | **0.64 [0.46, 0.89]** | **0.01** |
| Weekly household expenditure ^b^ | 1.01 [0.94, 1.08] | 0.79 |
| Number of people in household | 1.02 [0.99, 1.04] | 0.30 |
| Improved (cement/tile) floor material | 0.69 [0.42, 1.14] | 0.15 |

CI: Confidence Interval

^a^ Wealth quintile determined by assets owned by the household. The quintile ranges from poorest (1) to wealthiest (5).

^b^ Association reported per $10 USD spent.
