# Supplementary material for: Animal husbandry and environmental conditions are associated with cefotaxime-resistant Escherichia coli in yard soil in peri-urban Malawi
Source: PLOS Glob Public Health. 2026 Jul 13;6(7):e0006264. doi: 10.1371/journal.pgph.0006264 (PMC13362151; doi:10.1371/journal.pgph.0006264)
Supplement: S7 Table — Models include variables that were associated with the outcome with a p-value of <0.20 in bivariate analyses and also control for household’s primary water source, education, socioeconomics, household size, and indoor floor material. Bolded values indicate associations with p-value <0.05. (DOCX) [file pgph.0006264.s010.docx]

**S7 Table. Adjusted associations between household environmental characteristics, poultry ownership and prevalence of cefotaxime-resistant *E. coli* in yard soil.** Models include variables that were associated with the outcome with a p-value of <0.20 in bivariate analyses and also control for household’s primary water source, education, socioeconomics, household size, and indoor floor material. Bolded values indicate associations with p-value <0.05.

| Variable | Prevalence ratio (95% CI) | p-value |
| --- | --- | --- |
| Flush/pour flush | 0.90 [0.67, 1.21] | 0.48 |
| Latrine used by single household | 0.94 [0.75, 1.18] | 0.58 |
| Children openly defecate | 1.15 [0.94, 1.40] | 0.19 |
| Household owns poultry | 1.12 [0.94, 1.33] | 0.20 |
| Child used antibiotics in the last 4 weeks | **0.84 [0.70, 1.00]** | **0.05** |
| Soil in sunlight at time of collection | 0.97 [0.82, 1.15] | 0.71 |
| Soil dry at time of collection | **0.35 [0.25, 0.49]** | **<0.0005** |
| Ambient temperature top tertile | 0.93 [0.72, 1.21] | 0.60 |
| Ambient humidity top tertile | **0.84 [0.73, 0.97]** | **0.01** |
| Improved primary water source | 1.01 [0.84, 1.21] | 0.92 |
| Highest education in household is primary/incomplete secondary (vs. no formal education) | 0.89 [0.65, 1.22] | 0.47 |
| Highest education in household is secondary/post-secondary (vs. no formal education) | 0.94 [0.75, 1.18] | 0.60 |
| 2^nd^ Wealth quintile (vs. bottom quintile) ^a^ | 0.83 [0.65, 1.05] | 0.12 |
| 3^rd^ Wealth quintile (vs. bottom quintile) ^a^ | 0.81 [0.63, 1.03] | 0.08 |
| 4^th^ Wealth quintile (vs. bottom quintile) ^a^ | 0.80 [0.59, 1.09] | 0.16 |
| 5^th^ Wealth quintile (vs. bottom quintile) ^a^ | **0.62 [0.45, 0.85]** | **<0.005** |
| Weekly household expenditure ^b^ | 1.01 [0.94, 1.08] | 0.75 |
| Number of people in household | 1.02 [0.99, 1.05] | 0.30 |
| Improved (cement/tile) floor material | 0.69 [0.42, 1.14] | 0.15 |

CI: Confidence Interval

^a^ Wealth quintile determined by assets owned by the household. The quintile ranges from poorest (1) to wealthiest (5).

^b^ Association reported per $10 USD spent.
